# Is Dry Needling of the Supinator a Safe Procedure? A Potential Treatment for Lateral Epicondylalgia or Radial Tunnel Syndrome. A Cadaveric Study

**DOI:** 10.3390/ijerph18179162

**Published:** 2021-08-31

**Authors:** César Fernández-de-las-Peñas, Carlos López-de-Celis, Jacobo Rodríguez-Sanz, César Hidalgo-García, Joseph M. Donnelly, Simón A Cedeño-Bermúdez, Albert Pérez-Bellmunt

**Affiliations:** 1 Department of Physical Therapy, Occupational Therapy, Physical Medicine and Rehabilitation, Universidad Rey Juan Carlos (URJC), 28922 Alcorcón, Spain; 2 Cátedra Institucional en Docencia, Clínica e Investigación en Fisioterapia: Terapia Manual, Punción Seca y Ejercicio Terapéutico, Universidad Rey Juan Carlos, 28922 Alcorcón, Spain; 3 Faculty of Medicine and Health Sciences, Universitat Internacional de Catalunya (UIC-Barcelona), C/Josep Trueta s/n, Sant Cugat del Vallès, 08017 Barcelona, Spain; carlesldc@uic.cat (C.L.-d.-C.); jrodriguezs@uic.es (J.R.-S.); simoncedeno@uic.es (S.A.C.-B.); aperez@uic.es (A.P.-B.); 4 ACTIUM Functional Anatomy Group, 08195 Barcelona, Spain; 5 Unidad de Investigación en Fisioterapia, Universidad de Zaragoza, 50001 Zaragoza, Spain; hidalgo@unizar.es; 6 Department of Physical Therapy, Miami Campus, University of Saint Augustine for Health Sciences, Coral Gables, FL 33134, USA; donnelly.jm50@gmail.com

**Keywords:** supinator, dry needling, cadaver, safety, radial nerve, Frohse arcade

## Abstract

The supinator muscle is involved in two pain conditions of the forearm and wrist: lateral epicondylalgia and radial tunnel syndrome. Its close anatomical relationship with the radial nerve at the arcade of Frohse encourages research on dry needling approaches. Our aim was to determine if a solid filiform needle safely penetrates the supinator muscle during the clinical application of dry needling. Needle insertion of the supinator muscle was conducted in ten cryopreserved forearm specimens with a 30 × 0.32 mm filiform needle. With the forearm pronated, the needle was inserted perpendicular into the skin at the dorsal aspect of the forearm at a point located 4cm distal to the lateral epicondyle. The needle was advanced to a depth judged to be in the supinator muscle. Safety was assessed by measuring the distance from the needle to the surrounding neurovascular bundles of the radial nerve. Accurate needle penetration of the supinator muscle was observed in 100% of the forearms (needle penetration:16.4 ± 2.7 mm 95% CI 14.5 mm to 18.3 mm). No neurovascular bundle of the radial nerve was pierced in any of the specimen’s forearms. The distances from the tip of the needle were 7.8 ± 2.9 mm (95% CI 5.7 mm to 9.8 mm) to the deep branch of the radial nerve and 8.6 ± 4.3 mm (95% CI 5.5 mm to 11.7 mm) to the superficial branch of the radial nerve. The results from this cadaveric study support the assumption that needling of the supinator muscle can be accurately and safely conducted by an experienced clinician.

## 1. Introduction

The supinator is a deep forearm muscle that surrounds the proximal third of the radius. It is one of the major supinators of the forearm and assists with elbow flexion [1]. Further, it also plays a role in the lateral stability of the elbow [1]. Erak et al. suggested a biomechanical role for the superficial head of the supinator in the etiology of lateral epicondylalgia by inducing an increase in tensile force in the common wrist extensors tendon [2]. Others have proposed that the referred pain pattern elicited by the supinator muscle (e.g., primarily located to the lateral epicondyle and spreading to the dorsal aspect of the web of the thumb and the dorsal forearm) can contribute to symptoms experienced by people with lateral epicondylalgia [3]. In fact, injection of a hypertonic saline solution into the wrist extensor muscles, including the supinator muscle, mimicked sensorimotor features of lateral epicondylalgia [4].

A clinically relevant aspect of the supinator muscle is its close association with the radial nerve. This nerve can be entrapped at different anatomical locations throughout its course. An important, and sometimes underreported, potential entrapment area of the deep branch of the radial nerve is when it passes between the superficial and deep heads of the supinator, commonly referred to as the arcade of Frohse [5]. The compression of the posterior interosseous nerve at this location is called radial tunnel syndrome [6]. Symptoms of radial tunnel syndrome are similar than those experienced by patients with lateral epicondylalgia, e.g., deep aching pain in the lateral elbow and spreading dorsally and radially into the forearm [6]. These symptoms can increase with forearm rotation and lifting activities. Although first-line treatment of radial tunnel syndrome is conservative, there is a paucity in the literature regarding non-surgical treatment interventions for this syndrome [6].

Several interventions are promoted for managing muscle tissues and trigger points, e.g., manual therapy, exercise, physical agents, or dry needling [3]. Dry needling has received particular attention for practitioners in the last decade [7]. The American Physical Therapy Association (APTA) defines dry needling as a “skilled intervention using a thin filiform needle to penetrate the skin that stimulates myofascial trigger points, musculature and connective tissue for the management of neuromusculoskeletal disorders” [8]. In fact, different forms of application of dry needling are described in the literature [9]. Probably the most expanded needling approach used for the management of muscle trigger points is the “fast-in, fast-out” technique as described by Hong [9,10]. This approach consists of inserting the needle into a trigger point until a first local twitch response is obtained [10]. Once a first local twitch response is obtained, the needle is moved up and down, in and out of the trigger point to obtain more local twitch responses [10]. A recent meta-analysis has found low to moderate evidence suggesting a positive effect of dry needling for pain and disability in individuals with lateral epicondylalgia [11]. However, there is a paucity in the literature regarding specific data of needling the supinator muscle. Similarly, a recent case report described the successful management of radial tunnel syndrome with dry needling [12]. This case report observed immediate improvement in all outcomes after the first treatment session, with complete relief of pain symptoms six months following the fourth treatment session [12]. The potential effects of dry needling are explained by both mechanical and neurophysiological effects including a decrease in spontaneous electrical activity, disruption of contraction knots, increase muscular blood flow and oxygenation, a decrease of pro-inflammatory mediators, and stimulation of peripheral nerve fibers and the release of endogenous opioid and neurotransmitters [13].

The supinator muscle could contribute to forearm and/or wrist symptoms in two different ways. First, the pain referral from supinator muscle trigger points could directly reproduce the patient’s symptoms such as in lateral epicondylalgia. Second, an increased tension and shortening of the supinator muscle due to overuse or the presence of taut bands could increase the tension on the radial nerve and contribute to an entrapment such as in radial tunnel syndrome [3]. Therefore, application of dry needling would seem to be indicated for managing this muscle. Due to the anatomical relationship between the radial nerve and the supinator muscle, dry needling, although a potential effective treatment, is not exempt of risk of puncturing the radial neurovascular bundle. Although most adverse events experienced with dry needling applications are described as minor (e.g., bleeding, bruising, pain during the intervention), some major events (e.g., neuropraxia, numbness) can also occur [14]. McManus and Cleary described a neuropraxia of the radial nerve after application of dry needling at the junction of the middle and distal third of the humerus (where the radial nerve runs superficially) [15].

Anatomical landmarks represent the most common clinical method for applying dry needling. No anatomical study has investigated if a solid filiform needle, as used clinically with dry needling, accurately and safely penetrates the supinator muscle in relation to the superficial and deep branches of the radial nerve. The aim of this study was to determine the safety of dry needling the supinator muscle by measuring the distance between the needle and surrounding neurovascular bundles in a cadaver model.

## 2. Methods

### 2.1. Cadaveric Sample

Ten cryopreserved forearm cadaver specimens (4 females/5 males; 4 left/6 right forearms; mean age: 71, SD: 9 years; mean height: 162, SD: 10 cm; weight: 67.5, SD, 15.8 kg; forearm circumference: 22.2, SD: 3.2 cm) donated to the institutional university anatomy laboratory of a Universitat Internacional de Catalunya (Barcelona, Spain) were used for this study. The forearm specimens were visually checked for evidence of any structural abnormality, that would influence the anatomical study. The frozen forearms were stored at −20 °C and were thawed at room temperature 24 h prior to the study. The needling procedure was conducted with the forearm specimens unfrozen, but for the anatomical study, they were again frozen for better visualization of the tissues. This study was approved by the Local Ethics Committee of the Barcelona University (CBA-2020-2A).

### 2.2. Needling Procedure

**Figure 1:**
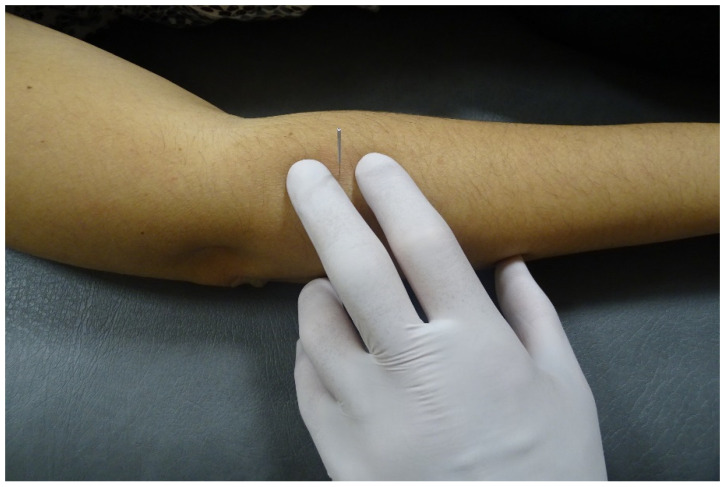
Illustration of needling insertion of the supinator muscle in a living individual.

The dry needling technique was conducted by a clinician with more than ten years of experience with dry needling. In this study, sterile stainless-steel filiform needles 30 mm in length and 0.32 mm caliber, with a plastic cylindrical guide, were used. The specimen forearm was placed in a pronated position. The filiform needle was inserted by the clinician in the upper third of the radius (dorsal aspect of the forearm) 4 cm distal to the lateral epicondyle in a dorsal to ventral direction (Figure 1). The needle was advanced toward the radius to a depth judged by the clinician to be in the supinator muscle.

### 2.3. Anatomical Procedure

**Figure 2:**
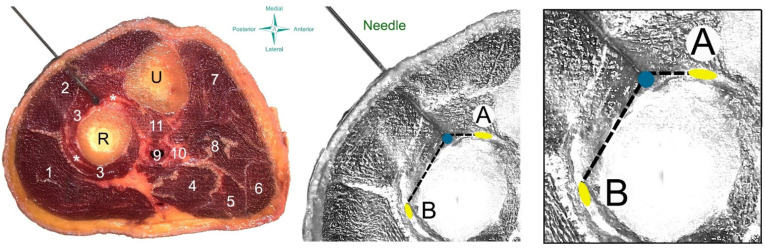
Scheme of the needling procedure of the supinator muscle on a cadaver (**left**) and anatomical draw (**center** and **right**). U: ulnar R: radio; 1: brachioradial muscle; 2 wrist extensor muscles; 3: supinator muscle; 4: pronator teres muscle; 5 flexor carpi radialis muscle; 6 palmaris longus muscle; 7: flexor carpi ulnaris muscle; 8; flexor digitorum superficialis muscle; 9 brachial vessels; 10 median nerve; 11: brachial muscle; * radial nerve (deep branch up and superficial branch down). A: deep branch of the radial nerve; B: superficial branch of the radial nerve.

Blue latex was injected to depict where the tip of the filiform needle was located to determine the accuracy of the insertion into the supinator. The needle was also left in situ during the anatomical procedure to assess the surrounding neurovascular bundles. Cross-sectional anatomical dissections of the upper third of the forearm were photographed and analyzed by photometry for measuring the following distances (Figure 2):

(1) Needle tip to the deep branch of the radial nerve (A): The distance (mm) between the tip of the needle and the deep branch of the radial nerve.
(2) Needle tip to the superficial branch of the radial nerve (B): The distance (mm) between the tip of the needle and the superficial branch of the radial nerve.

The needle penetration (the length of the needle inserted) for targeting the supinator muscle was also measured.

### 2.4. Imaging Procedure

Photographs were obtained with a digital camera (Cannon EOS1300D) oriented perpendicular to the forearm. The camera was held steady with a tripod during the imaging procedure. A millimeter ruler was placed next to the specimen to convert the measurements. The images were then analyzed using Adobe Photoshop CS6.

## 3. Results

### 3.1. General Findings

The dissection revealed that the tip of the needle pierced the supinator muscle belly in all forearms (accuracy 100%) after passing through the wrist extensor muscles. The needle was inserted a mean depth of 16.4 ± 2.7 mm (95% CI 14.5 mm to 18.3 mm) to reach the supinator (Figure 2 left image).

No neurovascular bundle was pierced during needling in any specimen forearms. The distances from the tip of the needle to the surrounding neurovascular bundles were 7.8 ± 2.9 mm (95% CI 5.7 mm to 9.8 mm) to the deep branch of the radial nerve (A) and 8.6 ± 4.3 mm (95% CI 5.5 mm to 11.7 mm) to the superficial branch of the radial nerve (B).

### 3.2. Findings by Gender

Female cadavers (*n* = 4) showed lower height, less weight and a smaller forearm circumference (height: 152, SD: 7 cm; weight: 50, SD, 5.7 kg; forearm circumference: 20.7, SD: 2.2 cm) than that of the male cadavers (*n* = 6; height: 169, SD: 5 cm; weight: 78, SD, 56.5 kg; forearm circumference: 23.1, SD: 3.5 cm).

The needle was inserted a mean depth of 16 ± 3 mm and of 17 ± 3 mm to reach the supinator in female and male cadavers, respectively. The distances from the tip of the needle to the surrounding neurovascular bundles were also similar between female (8 ± 4 mm to the deep branch -A- and 7 ± 3 mm to the superficial branch -B- of the radial nerve) and male (7 ± 3 mm to the deep branch -A- and 10 ± 5 mm to the superficial branch -B- of the radial nerve) cadavers.

## 4. Discussion

The results of this study found that dry needling, as applied in clinical practice, penetrates the supinator muscle with an accuracy of 100% when applied by an experienced clinician. The results also show utilizing this approach by using a standardized anatomical landmark that the needle did not pierce the superficial or deep branches of the radial nerve in all trials. Both branches of the radial nerve have an intimate anatomical relationship to the supinator muscle with the superficial radial nerve traversing over the muscle and the deep radial nerve passing through the arcade of Frohse (through the two heads) of the muscle. Dry needling of the supinator muscle with this approach provided enough space to avoid penetration of the deep or superficial (around 8 mm) branches of the radial nerve supporting the potential safety and efficacy of the procedure. A solid filiform needle has a gauze of 0.32 mm; therefore, these distances could be considered safe. In fact, these distances were similar between male and female cadavers, although this assumption must be considered with caution at this stage due to the small number of specimens used in the study. Additionally, clinicians should consider the presence of anatomical variations in the relationship between the supinator muscle and the deep branch of the radial nerve at the arcade of Frohse [6] and also anatomical variations in the superficial branch of the radial nerve [16,17]. The approach described in the current cadaveric study would probably be slightly different in clinical practice due to unexpected anatomical variations.

We found that the needle was inserted to a depth of 16.4 ± 2.7 mm to penetrate the supinator muscle, suggesting that no more than 20 mm of the needle should be inserted during clinical application of dry needling of this muscle. It should be noted that the tip of the needle reached the radius bone in eight out of ten of the specimens. There is evidence to support that the tip of the needle suffers slight deformation when impacting a bone [18]. Therefore, clinicians should control needle penetration when dry needling the supinator muscle to avoid hitting the radius. In such a scenario, anthropometric features of the patient could require different depths of needle insertion to reach the supinator muscle without impacting the radius bone. Therefore, our results should be considered according to the anthropometric features of the current forearm cadaver specimens. Interestingly, although male cadavers showed higher height and weight than female cadavers, the forearm circumference was similar between both genders. In fact, forearm circumference has been used to propose a prediction model for the selection of the needle length for the application of dry needling in the pronator teres muscle [19]. This study proposed a forearm circumference cut-off value of 27.5 cm to determine that the needle should be larger or not of 25 mm in length for preventing median nerve piercing during the application of dry needling [19]. Similarly, anthropometric features of the lower extremity have also been used for determining a prediction model for calculating the depth of the soleus muscle [20]. This study aimed to calculate the depth of the soleus muscle, and, therefore, the potential length of the needle for preventing tibial nerve piercing by crossing this muscle with the needle [20]. Due to the use of cadavers and the small number of specimens, we were not able to determine a prediction model for calculating the depth of the supinator muscle. Future “in vivo” studies using anthropometric features of the forearm will help to improve this knowledge.

The accuracy and safety of the placement of a solid filiform needle in the supinator muscle has implications for clinical practice since dry needling of the supinator muscle could be safely administered in patients with lateral epicondylalgia or radial tunnel syndrome. The results of this study should be considered by clinicians performing dry needling of the supinator muscle to avoid injury to the radial nerve. Additionally, clinicians should consider that the needle crosses the wrist extensor muscles before reaching the supinator muscle. These muscles must be considered in clinical trials that investigate the efficacy of dry needling in lateral elbow pain conditions. Nevertheless, it is important to determine that needling insertions in the current study were conducted by an experienced clinician. It is probably that safety and accuracy data of novice clinicians would be slightly different.

Some limitations of this investigation should be recognized. First, dissections were conducted in a small sample size (ten forearm specimens). Due to the small sample size, gender differences should be considered with caution at this stage. Gender differences in anthropometric data could influence the observed distances to the radial neurovascular bundle, although our preliminary results did not support this potential difference. Second, identification of anatomical landmarks is a requisite for a successful needle insertion. We used a standardized anatomical landmark for targeting the supinator muscle, however clinicians should consider that trigger point location in the supinator muscle may differ from the anatomical landmarks used in this study. Therefore, accuracy and safety of dry needling should be considered under the investigated conditions. Determining the accuracy and safety of inserting the needling in other areas of the supinator muscle will require future studies. Third, all needling insertions were conducted just once by an experienced clinician. We do not know the safety and accuracy of this dry needling approach when applied to viable tissues or by a novice clinician. Additionally, it would be really interesting to determine the intra- and inter-operator reliability of this needling procedure in future studies to assess differences between novice and experienced clinicians.

## 5. Conclusions

The results of this cadaveric study support that dry needling of the supinator muscle 4 cm distal to the lateral epicondyle of the humerus is an accurate and safe procedure when applied by an experienced clinician. Penetration of the radial neurovascular bundle was not observed in any trial utilizing a solid filiform needle of 30 × 0.32 mm when performed by an experienced clinician.

## Data Availability

All data derived from the study is available in the text.
